# Assessment of Associations Between Sociodemographic and Clinical Factors and Edentulism Complications in Patients Scheduled for Hybrid Prosthetic Therapy: A Cross-Sectional Study

**DOI:** 10.3390/clinpract15070133

**Published:** 2025-07-17

**Authors:** Shokraei Gholamreza, Doriana Agop-Forna, Cristina Dascălu, Norina Forna

**Affiliations:** Faculty of Dental Medicine, Grigore T. Popa University of Medicine and Pharmacy Iasi, Universitatii Street 16, 700115 Iasi, Romania; rochio1999@yahoo.com (S.G.); cristina.dascalu@umfiasi.ro (C.D.); norina.forna@umfiasi.ro (N.F.)

**Keywords:** edentulism, complications, implants, removable hybrid prostheses

## Abstract

Background/Objectives: Complications of edentulism include bone resorption, muscular dysfunction, temporomandibular joint disorders (TMJ), and stomatognathic system dysfunctional syndrome (SSDS). The objectives of the study were as follows: to analyze the distribution of edentulism complications related to sociodemographic and clinical parameters and to quantify the strength of relationships between edentulism complications and these socio-demographic or clinical variables. Materials and Methods: This cross-sectional study investigated 150 edentulous subjects (mean age 61.54 +/− 8.99 years) scheduled for hybrid prosthetic therapy. The distribution of edentulism complications was assessed in relation to sex-specific and age-specific patterns, edentulism location (maxillary vs. mandibular), edentulism extension (partial reduced, partial extended, subtotal, complete edentulism), and Kennedy classification (class I vs. class II vs. class IV). Cramér’s V was used to measure the strength of the association between edentulism complications and sociodemographic and clinical factors. Results: The most prevalent complications were more frequent in males—bone resorption (74.2% vs. 40.9%), malocclusion (97.5% vs. 84.9%), TMJ disorders (74.2% vs. 57.0%), muscular disorders (62.5% vs. 31.2%), dyshomeostasis (64.2% vs. 31.2%), and SSDS (79.2% vs. 53.8%). The most relevant associations were found between age group and clinical variables such as irregular ridge (Cramer’s V = 0.737), long/thick frenum (0.711), and SSDS (0.544), while edentulism category was strongly associated with irregular ridge (0.585), TMJ disorders (0.493), and bone resorption (0.492). Conclusions: The type and stage of edentulism emerged as key determinants of complication severity, with complete and subtotal edentulism being associated with the highest rates of muscular and temporomandibular joint dysfunctions.

## 1. Introduction

Partial and complete edentulism is a common condition in dental practice, with significant functional, aesthetic, and psychosocial implications for patients [1,2]. In the context of the development of implant-supported fixed prosthetics and the growing demand for stable rehabilitation solutions, hybrid prosthetic treatments are gaining increasing importance [3]. The rehabilitation of patients with partial or total edentulism using implant-supported hybrid prosthetic restorations significantly enhances quality of life by restoring optimal masticatory function and improving facial aesthetics. This treatment approach provides superior stability, comfort, and durability compared to conventional dentures, positively impacting the patient’s psychosocial well-being [4]. Also, the use of removable-implant-supported therapy in complete edentulism can avoid technical (fracture/chipping of the veneering ceramic, fractures of ceramic implant abutments, and aesthetic problems) specific to implant-retained multiple-unit fixed-prosthetic restorations [5,6].

Effective treatment planning requires a thorough assessment of pre-prosthetic and pre-implant complications, such as alveolar bone resorption, irregular residual ridges, bulky frenula, malocclusion, temporomandibular disorders, and muscular dysfunctions [7,8]. In the case of pre-prosthetic or pre-implant complications like bone resorption, residual ridge irregularities, or temporomandibular joint dysfunctions, the choice of the best prosthetic solution is important for treatment success. Thus, due to the advantages and disadvantages of fixed- and removable-implant-supported prosthesis, the choice between them should be carefully weighed. Although fixed prostheses provide better stability and aesthetic results, removable prostheses can limit biomechanical strain and technical problems, particularly in cases with advanced alveolar bone resorption or morphological constraints. As pointed out by Messias et al., a comparison of these prosthetic approaches is very important to obtain a personalized treatment planning to edentulous maxilla [9]. Edentulism complications may influence the prognosis of oral rehabilitation, and factors such as design, materials, patient education, and compliance to follow-up influence treatment success. All these factors lead to a percentage of almost 40% of patients that no longer use their removable partial dentures at 5 years post-treatment due to pain and aesthetic disorders [10]. Long-term outcome can be improved by implant-assisted hybrid prosthetic restorations as well as new concepts and framework design considerations and technological and materials advancements in the field [11]. From this point of view, it is necessary to consider how long-term implant survival is offered by the mucosal seal, which, depending on the prosthetic components used, allows for an increase in the stability of the peri-implant tissues over time [12]. Intraoral edentulous complications are mainly represented by alveolar bone resorption and uneven residual ridges. Edentulism is also a factor in malocclusion and disturbances of cranio-mandibular alignment [13]. Hybrid prosthetic restoration becomes greatly problematic in patients with malocclusion, mandibular latero-deviations, temporomandibular joint (TMJ) problems (temporomandibular joint pain and muscular dysfunction), and mandibulo-cranial (MC) misalignment. Lack of stable and multiple dental contacts can lead to a reduction in the vertical dimension of the lower face [14]. TMJ dysfunction and masticatory muscle disorders can lead to functional limitations in mouth opening and mandibular deviation [15]. Consequently, treatment planning should consider occlusal relationships as a co-factor in the development of various pathologies of the stomatognathic system. Although occlusal factors alone rarely cause temporomandibular disorders (TMD) or peri-implant bone loss, they may exacerbate functional overload and tissue breakdown, especially when combined with inflammatory or mechanical stressors [16,17]. Since the components of the stomatognathic system are highly interconnected, pathological changes in one structure often result in dysfunction and pathologies in other structures. Dysfunction of this system is particularly exacerbated through cranio-mandibular misalignment, and such misalignment can drastically reduce therapeutic options [18]. Specialists in prosthetics and implantology need an in-depth understanding of the case to address all complications in order to achieve stability of the future prosthetic restoration and muco-osseous support [19,20].

The aim of this study was to analyze the prevalence and distribution of edentulism complications in relation to sociodemographic and clinical parameters of edentulous patients eligible for hybrid prosthetic therapy.

## 2. Materials and Methods

### 2.1. Study Design

The study group included 150 edentulous subjects (sex: 82 males, 68 females; mean age 61.54 ± 8.99 years) scheduled for hybrid prosthetic therapy in the Clinical Base of Dental Medicine Faculty, U.M.F. “Grigore T. Popa”, Iași, between 30 February 2023 and 30 May 2024. Inclusion criteria were as follows: (i) age over 18 years old, (ii) partial, subtotal, or complete edentulism in at least one arch, and (iii) eligible for teeth- or implant-supported hybrid prosthetic rehabilitation. Exclusion criteria were as follows: active oral infections, recent maxillofacial trauma history, systemic conditions that contraindicate implant placement, and incomplete clinical documentation.

The research was conducted in accordance with the Declaration of Helsinki and approved by the Institutional Review Board (or Ethics Committee) of U.M.F. “Grigore T.Popa”, Iasi (Romania) (no 413, approved on 11 March 2024). Informed consent was obtained from all subjects involved in the study.

### 2.2. Diagnosis of Edentulism Complications

Two calibrated evaluators performed clinical and radiological examinations of the patients in the study group to detect edentulism complications in the pre-prosthetic and pre-implant phases. Pre-prosthetic complications refer to clinical issues that must be addressed prior to the planning or execution of prosthetic treatment. Pre-implant complications refer to conditions that may affect implant placement. These edentulism complications include alveolar bone atrophy, mandibular deviations, temporomandibular joint disorders, and related functional impairments.

The Cawood and Howell classification was employed to assess alveolar ridge resorption, with data collection focusing specifically on the presence of resorption corresponding to class IV (moderate resorption) and class V (severe resorption) based on the observed reduction in ridge height and width during clinical and CBCT evaluations. This method provided a reliable estimation of bone loss, ensuring consistency and reproducibility in distinguishing between severe and non-severe resorption cases.

Both the local and loco-regional evaluation of the stomatognathic system dysfunction syndrome (SDSS) and temporomandibular disorders (TMD) examined the temporomandibular joints (TMJ), the masticatory muscles, and the mandibulo-cranial relationships. A full medical and dental history was taken as part of the diagnostic plan, including the onset, duration, and quality of the pain, as well as any potential risk factors such as trauma, bruxism, and psychological stress. Clinical examination included static and dynamic functional examination of the TMJ, palpation for muscle tenderness, joint noise, occlusion, and intermaxillary relations [21,22,23]. Imaging and functional tests (ultrasonography, cone beam computed tomography (CBCT)) were performed as paraclinical investigations, particularly in the case of suspected malocclusion or intra-articular pathology [22], to identify TMJ alterations concerning improper condyle–disc relationships, inflammatory conditions (arthritis), degenerative joint modifications (osteoarthritis), or trauma [23]. TMD was diagnosed according to the established criteria [13,24]:

- Myalgias were as follows: myalgia, local myalgia, myofascial pain, referred myofascial pain, arthralgia, and headache attributed to TMD;
- Non-painful internal derangements were as follows: disc displacement with reduction, with reduction and intermittent locking, without reduction and with limited opening, and without reduction and without limited opening, degenerative joint disease, and subluxation;
- Less frequent diseases were as follows: fractures of the TMJ, manifestations of systemic disease, neoplasms, and developmental malformations.

According to DC/TMD criteria [13], diagnosis was made based on clinical symptoms as well as objective findings. Single signs unassociated with pain or mandibular dysfunction were not used to make a diagnosis of TMD.

The following variables were described for each patient:

- Age and sex;
- Partial edentulism (extended partial edentulism—EPE; reduced partial edentulism—RPE; subtotal edentulism—SE; complete edentulism—CE);
- Edentulism location related to arch involved (maxilla—MX, mandible—MD, or both—MX/MD);
- Kennedy classification of edentulism;
- Pre-prosthetic/pre-implant complications:
- Irregular residual ridge;
- Atrophy of the alveolar bone;
- Bulky, enlarged, or unfavorably positioned frenulum;
- Malocclusion;
- TMJ disorders (pain, TMJ noises);
- Mandibular–cranial (MC) misalignment;
- Muscular dysfunction;
- Stomatognathic system dysfunctional syndrome (SSDS).

### 2.3. Data Analysis

The necessary sample size was calculated using the GPower 3.1 software, assuming a medium effect size of 0.3, a standard alpha error 0.05, and a standard power of 0.8 (for chi-squared with 3 degrees of freedom, the minimal required sample size was 122 patients; for chi-squared with 2 degrees of freedom, the minimal required sample size was 108 patients; for chi-squared with 1 degree of freedom, the minimal required sample size was 88 patients). Statistical analyses were conducted using Microsoft Excel and SPSS 29.0. Nominal variables (sex, age groups, location, edentulism extension, Kennedy class) were described by frequencies, and their relationships were tested using the χ^2^ test and Cramer’s V association coefficient. Cramer’s V was used to measure of the strength of the association between two categorical variables in a contingency table. It is particularly used to evaluate relationships between variables when both are nominal. Low values (close to 0) indicate a weak or nonexistent association between variables. Moderate values (0.1–0.3) suggest a moderate association that may be clinically relevant. High values (above 0.3) indicate a strong association that is statistically significant and potentially clinically relevant. Differences in the prevalence and distribution of edentulism complications were analyzed in relation to sex, age groups, type of edentulism (extension, Kennedy class), and arch involvement. The significance level was set to *p* < 0.05.

## 3. Results

Table 1 shows the features of the study group. Table 1 shows the socio-demographic and edentulism parameters of the subjects included in the study group. Clinical and paraclinical evaluations were performed for each patient to detect and assess edentulism complications. The study sample consisted of 45.3% female and 54.7% male patients. In terms of age distribution, 8.7% were aged between 41 and 50 years, 33.3% were between 51 and 60 years, 40.0% were between 61 and 70 years, and 18.0% were between 71 and 80 years. Regarding edentulism parameters, 23.3% of the patients were mandibular edentulous, 34.7% were maxillary edentulous, and 42.0% were edentulous in both arches. As for the type of edentulism, EPE was the most common, found in 56.7% of cases, followed by CE in 31.3%, while RPE and SE each accounted for 6.0% of the patients. Analyzing the distribution according to Kennedy classification, class I was present in 34.4% of cases, followed by class I + I in 31.3%, class I + II and class II each in 12.5%, and class I + IV in 6.3%

In a previous study, we assessed the prevalence rates of each edentulism complication in patients eligible for implant-supported hybrid prosthetic therapy [25]. Among the studied patients, the most prevalent complication was malocclusion (88.7%). Temporomandibular disorders were also common (68.7%), while maxillomandibular misalignments were identified in 65.3% of cases. Medium-to-advanced bone resorption was present in 63.3% of patients, and an irregular alveolar ridge was reported in 41.3% of cases. Muscular disorders were noted in 48.7% of patients, whereas abnormalities of the frenulum were found in 10.0% of the patients [25]. In this study, we focused on the prevalence and distribution of edentulism complications in relation to sex-specific and age-specific patterns, edentulism location (maxillary vs. mandibular), edentulism extension (partial reduced edentulism, partial extended edentulism, subtotal edentulism, complete edentulism), and Kennedy classification of edentulism (class I vs. class II vs. class IV). We also evaluated the strength of the association between each type of edentulism complication and the assessed socio-demographic and clinical factors.

### 3.1. Sex-Specific Patterns in Oral and Functional Complications of Edentulism

Table 2 shows the distribution of edentulism complications related to sex. Statistically significant associations between gender and several clinical variables were identified. Bone resorption was more frequent in males compared to females (*p* < 0.001). A long or thick frenum was present exclusively in males (*p* < 0.001). Malocclusion was also more prevalent in males than in females (*p* = 0.001). Muscular disorders were reported more often in males than in females (*p* < 0.001), and midline cranial misalignment (MC misalignment) was more frequent in males (*p* = 0.004). Dyshomeostasis occurred more frequently in males (*p* < 0.001), and SSDS was more commonly reported in males (*p* = 0.002).

The strongest associations with gender were observed for dyshomeostasis (Cramer’s V = 0.315), long/thick frenum (0.304), muscular disorders (0.297), and bone resorption (0.280), all reflecting moderate effect sizes. Other variables showed weaker associations, with Cramer’s V values below 0.30, suggesting a minimal-to-low association strength.

### 3.2. Age-Specific Patterns in the Mouth and Functional Complications of Edentulism

Table 3 shows the distribution of edentulism complications related to age groups. Statistically significant associations were found between age group and several clinical parameters. Bone resorption increased markedly with age (*p* < 0.001). Irregular ridge showed a similar trend, being more frequent in older age groups (*p* < 0.001). A long or thick frenum was observed only in the oldest group (*p* < 0.001). TMJ disorders became progressively more frequent with age (*p* < 0.001). Muscular disorders also increased significantly with age (*p* < 0.001). MC misalignment showed a rising trend with advancing age (*p* < 0.001). TMJ pain peaked in the middle age groups and was absent in the youngest (*p* < 0.001). Dyshomeostasis was more frequent with increasing age (*p* = 0.001). SSDS followed a similar pattern, being more prevalent in the older groups (*p* < 0.001). The strongest associations with age group were observed for irregular ridge (Cramer’s V = 0.737), long/thick frenum (0.711), SSDS (0.544), and TMJ pain (0.505), indicating very-strong-to-strong effect sizes. Moderate associations were found for muscular disorders (0.453), bone resorption (0.451), TMJ disorders (0.439), MC misalignment (0.384), and dyshomeostasis (0.324), while malocclusion showed only a weak association (0.216).

### 3.3. Edentulism Location Patterns in the Mouth and Functional Complications of Edentulism

Table 4 shows the distribution of edentulism complications related to arches (mandibular vs. maxillary). Statistically significant associations were observed between dental arch and several clinical variables. Irregular ridge was more frequently found in the mandibular arch (*p* = 0.001). A long or thick frenum was significantly more common in the mandibular arch (*p* < 0.001). Additionally, malocclusion was more prevalent in the maxillary arch (*p* = 0.005). The strongest associations with dental arch were found for long/thick frenum (Cramer’s V = 0.289), irregular ridge (0.273), and malocclusion (0.250), indicating moderate effect sizes. All other variables showed weak or negligible associations, with Cramer’s V values below 0.20.

### 3.4. Edentulism Extension Patterns in the Mouth and Functional Complications of Edentulism

Table 5 shows the distribution of complications related to edentulism extension. Bone resorption was significantly associated with the type of edentulism (*p* < 0.001). Irregular ridge showed a significant relationship with edentulism category (*p* < 0.001). A long or thick frenum was also significantly associated with the type of edentulism (*p* < 0.001). Malocclusion was significantly more prevalent depending on the edentulism type (*p* < 0.001). TMJ disorders were strongly associated with edentulism category (*p* < 0.001). Muscular disorders showed a significant correlation with the type of edentulism (*p* < 0.001). Midline cranial misalignment was significantly related to edentulism type (*p* < 0.001). TMJ pain demonstrated a significant association with specific edentulism category (*p* < 0.001). Dyshomeostasis was significantly linked to edentulism type (*p* < 0.001). SSDS was strongly associated with the type of edentulism (*p* < 0.001). The strongest associations between edentulism category and clinical variables were observed for irregular ridge (Cramer’s V = 0.585), temporomandibular joint (TMJ) disorders (0.493), muscular disorders (0.493), bone resorption (0.492), malocclusion (0.482), midline cranial misalignment (0.481), and SSDS (0.486), indicating strong effect sizes.

### 3.5. Kennedy Classification

Table 6 shows the distribution of edentulism-related complications according to Kennedy class. Statistically significant differences between Kennedy class and clinical variables were found for TMJ disorders, muscular disorders, and TMJ pain. TMJ disorders were significantly associated with Kennedy classification (*p* = 0.021). Muscular disorders showed a borderline significant relationship with Kennedy class (*p* = 0.050). TMJ pain demonstrated a strong and significant association with Kennedy classification (*p* = 0.003). The strongest association was observed for TMJ pain (Cramer’s V = 0.351), followed by TMJ disorders (0.283) and muscular disorders (0.250), all indicating moderate effect sizes.

## 4. Discussion

The main objective of our study was to investigate the prevalence and distribution of complications in edentulous patients scheduled for hybrid prosthetic therapy. We also aimed to quantify the strength of the relationships between edentulism complications and these clinical or socio-demographic variables. This provides a basis for identifying relevant risk factors and guiding clinical interventions or prevention strategies. Understanding these complications is essential for optimizing treatment protocols, minimizing failure risks, and ensuring long-term functional and aesthetic outcomes in complex prosthetic rehabilitations [26,27,28,29].

### 4.1. Sex-Specific Patterns in the Mouth and Functional Complications of Edentulism

Several statistically significant sex differences were observed in the prevalence of specific complications. Bone resorption was significantly more frequent in males compared to females (*p* < 0.001 **), suggesting that male patients may be more prone to advanced alveolar bone loss prior to prosthetic treatment. A long or thick frenulum was reported only in males, with no cases observed in females (*p* < 0.001 **), pointing to a sex-related anatomical predisposition requiring surgical correction before prosthetic procedures in males. Malocclusion was significantly more common in males than females (*p* < 0.001 **), with males showing a higher need for occlusal adjustment or corrective therapy. TMJ disorders were highly significantly more frequent in males versus females (*p* = 0.008 **), a highly significant difference that reflects sex-specific biomechanical or parafunctional habits contributing to temporomandibular joint dysfunction. Muscular disorders were also significantly more prevalent in males (*p* = 0.001 **), reinforcing the potential link between male patients and increased muscular strain or imbalances during mastication or parafunctions. Maxillocranial misalignment showed a significantly higher prevalence in males than females (*p* = 0.001 **), highlighting the importance of occlusal and skeletal relationship assessment in male patients during treatment planning. Dyshomeostasis was notably more frequent in males versus females (*p* < 0.001 **), suggesting that neuromuscular imbalance is more commonly encountered in male patients. Differences in SSDS prevalence between males and females (*p* < 0.001 **) emphasize that male patients may be at greater risk for stomatognathic system dysfunction syndromes associated with poor oral status. The higher frequency of complications in male patients may reflect differences in oral hygiene behavior, bruxism prevalence, or delayed treatment-seeking patterns [17,25,30].

### 4.2. Age-Specific Patterns in the Mouth and Functional Complications of Edentulism

In the analysis of age-related differences in edentulism complications, several highly significant statistical associations were observed. Bone resorption showed a progressive increase with age, a highly significant trend (*p* < 0.001 **). Irregular alveolar ridge in various age groups indicates an age-related deterioration of ridge morphology, likely due to prolonged edentulism or resorption processes. The presence of a long or thick frenulum in various age groups (*p* < 0.001 **) indicates a highly significant difference reflecting age-related soft tissue changes or adaptation mechanisms in edentulous patients. Malocclusion was frequent across all age groups, with no statistically significant differences (*p* = 0.085). TMJ disorders showed a steep age-related increase (*p* < 0.001 **). Muscular disorders were distributed across age groups (*p* < 0.001 **). Maxillocranial misalignment showed a highly significant variation, emphasizing the need for skeletal and occlusal assessment in older adults (*p* < 0.001 **). TMJ pain had an uneven distribution, which may reflect transient pain episodes or age-specific adaptive responses (*p* < 0.001 **). Dyshomeostasis followed a rising trend (*p* < 0.001 **), supporting the idea that local and loco-regional instability may increase with age. The SSDS distribution across age groups suggests an age-dependent accumulation of somatic dysfunction patterns potentially linked to chronic oral changes. Age-related trends may also be influenced by cumulative bone loss, systemic conditions, or reduced adaptive capacity of the masticatory system in older individuals, which can compromise prosthetic success [17,25,30].

### 4.3. Edentulism Location Patterns in the Mouth and Functional Complications of Edentulism

When comparing the distribution of complications between the maxillary and mandibular arches, no statistically significant differences were found, suggesting that the location of edentulism does not meaningfully impact the occurrence of pre-prosthetic or pre-implant complications. Bone resorption tended to be slightly more frequent in the maxillary arch, while a long or thick frenulum appeared more often in the mandible, but neither difference was statistically significant (*p* = 0.072 and *p* = 0.066, respectively). Malocclusion was common in both arches (*p* = 0.550), and TMJ disorders, muscular issues, maxillocranial misalignments, and TMJ pain showed very similar rates regardless of arch location. Likewise, dyshomeostasis and SSDS were evenly distributed. Overall, these findings suggest that whether tooth loss occurs in the upper or lower jaw, it does not significantly influence the type or frequency of related complications [17,25,30].

### 4.4. Edentulism-Extension-Specific Patterns in the Mouth and Functional Complications of Edentulism

The analysis of edentulism-related complications across different categories of edentulism extension indicated a strong association between the clinical stage of edentulism and the presence of edentulism complications. The bone resorption distribution reflects the progressive and cumulative nature of alveolar bone loss over time. An irregular alveolar ridge emphasizes the anatomical remodeling associated with long-standing edentulism. The malocclusion distribution indicates that occlusal dysfunction becomes more prominent as edentulism evolves or stabilizes. TMJ disorders followed a similar trend, highlighting the biomechanical consequences of prolonged occlusal imbalance. The muscular disorders distribution may indicate an increased neuromuscular compensation or dysfunction in complete or subtotal edentulism. TMJ pain had a selective distribution linked to individual variations in functional adaptation or chronicity of joint overload. The dyshomeostasis distribution suggests that loco-regional imbalance may develop over time with chronic evolution and is absent in early cases of edentulism. Thus, the distribution patterns of malocclusion, TMJ disorders, muscular disorders, TMJ pain, dyshomeostasis, and SSDS suggest that as edentulism progresses or stabilizes, occlusal dysfunction and neuromuscular imbalance become more pronounced, likely due to prolonged biomechanical stress and adaptive limitations, with SSDS (*p* < 0.001) further reinforcing the link between the chronicity of edentulism and the development of somatic dysfunction syndromes [17,25,30].

### 4.5. Edentulism Kennedy Classification Patterns in the Mouth and Functional Complications of Edentulism

In the analysis of complications related to edentulism Kennedy classification, most parameters did not show statistically significant differences, with the exception of TMJ pain and SSDS. The bone resorption distribution showed differences that were not statistically significant (*p* = 0.158), suggesting that bone loss may occur irrespective of Kennedy classification. Irregular alveolar ridge was observed to have a distribution with differences that were not statistically relevant (*p* = 0.315). No cases of a long or thick frenulum were reported in any group, indicating uniformity for this parameter across Kennedy classifications. Malocclusion affected the majority of patients, but although class IV patients had the highest prevalence, the difference was not statistically significant (*p* = 0.205), possibly due to the small sample size in that category. Temporomandibular joint disorders were more prevalent in class IV compared to class I and class II, with this difference suggesting a trend. Muscular disorders distribution differences were not statistically significant (*p* = 0.107), though the distribution suggests higher muscle involvement in more extensive edentulism (classes I and IV). Maxillocranial misalignment indicates a lack of substantial variation in interarch discrepancies across classes (*p* = 0.693). The TMJ pain distribution suggests that patients with anterior single tooth loss (class IV) may be at significantly higher risk for TMJ pain, possibly due to altered loading or compensatory function. The dyshomeostasis distribution had no significant differences, indicating that loco-regional imbalance may not be directly related to the classification of partial edentulism. The SSDS distribution suggests that the extent and location of edentulism may be associated with a greater tendency toward loco-regional dysfunction, particularly in anterior or bilateral edentulism patterns. The predominance of Kennedy class I and II cases among those requiring removable prosthetic solutions aligns with previous studies emphasizing the biomechanical challenges associated with distal extension edentulism. Our results suggest that such configurations remain clinically demanding due to uneven load distribution and the need for optimized support from both implants and soft tissues [17,25,30].

### 4.6. Practical Implications

While the distribution of complications is consistent with patterns observed in similar descriptive studies [17,25,28,29,30], our dataset adds to the literature by combining both fixed- and removable-implant-supported rehabilitations in the same clinical framework. This may guide treatment planning toward more individualized approaches, considering anatomical, functional, and patient-related variables. Clinically, these results support the need for thorough diagnostic work-ups, including functional occlusal analysis and muscular assessment, especially in patients with previous prosthetic failures or signs of stomatognathic system dysfunction. Considering the interconnected nature of the stomatognathic system, dysfunction in one component frequently disrupts others, with mandibular–cranial misalignment intensifying the severity of these issues. This exacerbation complicates mandibular recovery and may, in some cases, restrict therapeutic options [15].

A research group reported that missing teeth, occlusal relationships, periodontal health of the remaining teeth, and the cranio-mandibular relationship are considered local factors influencing the balance of the stomatognathic system, while other factors include age, neuromuscular control, mental status, and overall health resilience [28]. A multifactorial theory must be considered to explain TMJ disorders, and possible etiology must be analyzed from different aspects [29]. Thus, the management of the extensive partial edentulism as well as a subtotal or complete edentulous arch requires an in-depth understanding of the vertical and horizontal restorative space needed for various types of hybrid prostheses to avoid instability, aesthetic compromise, and improper contours of the future denture [28]. The assessed parameters significantly influence TMJ status, a mechanically demanding and biochemically dynamic environment [30,31,32,33,34,35,36]. In-depth knowledge about the prevalence and distribution of edentulism complications highlights the need for early detection and personalized treatment of TMJ dysfunction and muscular disorders, especially among older and male patients, in order to improve the stability of future prosthodontic restorations [32,33]. This study underlines the importance of a multidisciplinary analysis of edentulous patients, also correlating occlusal, skeletal, and neuromuscular information, in order to avert treatment failure.

### 4.7. Limitations

The cross-sectional nature of the study is a limitation, as it does not allow for the development of causal relationships or the assessment of the progression of complications over time. The sample being obtained from a single academic institution may restrict the generalizability of the findings to other populations or clinical settings. The absence of functional imaging or reliable neuromuscular tests might underestimate or overlook more subtle dysfunctions, especially in early-stage cases. Some subgroups, such as patients with Kennedy class IV edentulism, had small sample sizes, and as a result, there may be insufficient statistical power to detect significant differences. Systemic diseases, medication use, and previous prosthetic history could have acted as confounding variables in some complications and were not considered in the analysis. Further prospective studies with larger and more balanced samples are necessary to confirm these preliminary observations and to better understand the impact of edentulous classification and local anatomical factors on long-term implant prosthetic outcomes.

## 5. Conclusions

The type and stage of edentulism emerged as key determinants of complication severity, with complete and subtotal edentulism being associated with the highest rates of muscular and temporo-mandibular joint dysfunctions. Gender was moderately associated with factors like dyshomeostasis, a long or thick frenum, muscular disorders, and bone resorption, while other variables showed only weak links. Age group had the strongest connections to irregular ridge, a long or thick frenum, SSDS, and TMJ pain, with moderate associations for muscular and TMJ disorders, bone resorption, and other factors, emphasizing its substantial impact on clinical outcomes.

## Figures and Tables

**Table 1:** Features of the edentulous patients scheduled for hybrid prosthetic therapy.

		Total	
		N	%
Demographics			
Sex	F	68	45.3%
	M	82	54.7%
Age groups	41–50	13	8.7%
	51–60	50	33.3%
	61–70	60	40.0%
	71–80	27	18.0%
Edentulism parameters			
Edentulous arch	MD	35	23.3%
	MX	52	34.7%
	MX + MD	63	42.0%
Edentulism type	PEE	85	56.7%
	PRE	9	6.0%
	CE	47	31.3%
	SE	9	6.0%
Kennedy class	I	33	34.4%
	I + I	30	31.3%
	I + II	12	12.5%
	I + IV	6	6.3%
	II	12	12.5%
	II + II	3	3.1%
Complete edentulism		54	64%

PEE—partial extended edentulism; PRE—partial reduced edentulism; CE—complete edentulism; SE—subtotal edentulism.

**Table 2:** Distribution of edentulism complications by gender in patients eligible for hybrid prosthetic therapy.

		Gender				Total		Pearson’s Chi-Squared Test (df = 1)	Cramer’s V
		F		M					
		N	%	N	%	N	%		
Bone resorption	Yes	33	48.5%	62	75.6%	95	63.3%	Chi^2^ = 11.739	0.280 **
	No	35	51.5%	20	24.4%	55	36.7%	*p* < 0.001 **	
Irregular ridge	Yes	27	39.7%	35	42.7%	62	41.3%	Chi^2^ = 0.136	0.030
	No	41	60.3%	47	57.3%	88	58.7%	*p* = 0.712	
Long/thick frenum	Yes	-	-	15	18.3%	15	10.0%	Chi^2^ = 13.821	0.304 **
	No	68	100.0%	67	81.7%	135	90.0%	*p* < 0.001 **	
Malocclusion	Yes	54	79.4%	79	96.3%	133	88.7%	Chi^2^ = 10.603	0.266 **
	No	14	20.6%	3	3.7%	17	11.3%	*p* = 0.001 **	
TMJ disorders	Yes	41	60.3%	59	72.0%	100	66.7%	Chi^2^ = 2.273	0.123
	No	27	39.7%	23	28.0%	50	33.3%	*p* = 0.132	
Muscular disorders	Yes	22	32.4%	51	62.2%	73	48.7%	Chi^2^ = 13.251	0.297 **
	No	46	67.6%	31	37.8%	77	51.3%	*p* < 0.001 **	
MC misalignment	Yes	36	52.9%	62	75.6%	98	65.3%	Chi^2^ = 8.434	0.237 **
	No	32	47.1%	20	24.4%	52	34.7%	*p* = 0.004 **	
TMJ pain	Yes	9	13.2%	21	25.6%	30	20.0%	Chi^2^ = 3.558	0.154
	No	59	86.8%	61	74.4%	120	80.0%	*p* = 0.059	
Dyshomeostasis	Yes	20	29.4%	50	61.0%	70	46.7%	Chi^2^ = 14.880	0.315 **
	No	48	70.6%	32	39.0%	80	53.3%	*p* < 0.001 **	
SSDS	Yes	38	55.9%	65	79.3%	103	68.7%	Chi^2^ = 9.449	0.251 **
	No	30	44.1%	17	20.7%	47	31.3%	*p* = 0.002 **	
Total		68	100.0%	82	100.0%	150	100.0%		

** highly statistically significant.

**Table 3:** Distribution of edentulism complications by age group in patients eligible for hybrid prosthetic therapy.

		Age Group								Total		Pearson’s Chi-Squared Test (df = 3)	Cramer’s V
		41–50		51–60		61–70		71–80					
		N	%	N	%	N	%	N	%	N	%		
Bone resorption	Yes	2	15.4%	27	54.0%	39	65.0%	27	100.0%	95	63.3%	Chi^2^ = 30.449	0.451 **
	No	11	84.6%	23	46.0%	21	35.0%	-	-	55	36.7%	*p* < 0.001 **	
Irregular ridge	Yes	-	-	-	-	38	63.3%	24	88.9%	62	41.3%	Chi^2^ = 81.543	0.737 **
	No	13	100.0%	50	100.0%	22	36.7%	3	11.1%	88	58.7%	*p* < 0.001 **	
Long/thick frenum	Yes	-	-	-	-	-	-	15	55.6%	15	10.0%	Chi^2^ = 75.926	0.711 **
	No	13	100.0%	50	100.0%	60	100.0%	12	44.4%	135	90.0%	*p* < 0.001 **	
Malocclusion	Yes	13	100.0%	42	84.0%	51	85.0%	27	100.0%	133	88.7%	Chi^2^ = 6.999	0.216
	No	-	-	8	16.0%	9	15.0%	-	-	17	11.3%	*p* = 0.072	
TMJ disorders	Yes	3	23.1%	27	54.0%	43	71.7%	27	100.0%	100	66.7%	Chi^2^ = 28.900	0.439 **
	No	10	76.9%	23	46.0%	17	28.3%	-	-	50	33.3%	*p* < 0.001 **	
Muscular disorders	Yes	2	15.4%	15	30.0%	32	53.3%	24	88.9%	73	48.7%	Chi^2^ = 30.746	0.453 **
	No	11	84.6%	35	70.0%	28	46.7%	3	11.1%	77	51.3%	*p* < 0.001 **	
MC misalignment	Yes	2	15.4%	30	60.0%	42	70.0%	24	88.9%	98	65.3%	Chi^2^ = 22.140	0.384 **
	No	11	84.6%	20	40.0%	18	30.0%	3	11.1%	52	34.7%	*p* < 0.001 **	
TMJ pain	Yes	-	-	24	48.0%	6	10.0%	-	-	30	20.0%	Chi^2^ = 38.250	0.505 **
	No	13	100.0%	26	52.0%	54	90.0%	27	100.0%	120	80.0%	*p* < 0.001 **	
Dyshomeostasis	Yes	-	-	24	48.0%	28	46.7%	18	66.7%	70	46.7%	Chi^2^ = 15.750	0.324 **
	No	13	100.0%	26	52.0%	32	53.3%	9	33.3%	80	53.3%	*p* = 0.001 **	
SSDS	Yes	-	-	30	60.0%	46	76.7%	27	100.0%	103	68.7%	Chi^2^ = 44.340	0.544 **
	No	13	100.0%	20	40.0%	14	23.3%	-	-	47	31.3%	*p* < 0.001 **	
Total		13	100.0%	50	100.0%	60	100.0%	27	100.0%	150	100.0%		

** highly statistically significant.

**Table 4:** Distribution of edentulism complications by edentulism location in patients eligible for hybrid prosthetic therapy.

		Arch				Total		Pearson’s Chi-Squared Test (df = 1)	Cramer’s V
		MD		MX					
		N	%	N	%	N	%		
Bone resorption	Yes	20	57.1%	75	65.2%	95	63.3%	Chi^2^ = 0.753	0.071
	No	15	42.9%	40	34.8%	55	36.7%	*p* = 0.385	
Irregular ridge	Yes	23	65.7%	39	33.9%	62	41.3%	Chi^2^ = 11.191	0.273 **
	No	12	34.3%	76	66.1%	88	58.7%	*p* = 0.001 **	
Long/thick frenum	Yes	9	25.7%	6	5.2%	15	10.0%	Chi^2^ = 12.526	0.289 **
	No	26	74.3%	109	94.8%	135	90.0%	*p* < 0.001 **	
Malocclusion	Yes	26	74.3%	107	93.0%	133	88.7%	Chi^2^ = 9.395	0.250 **
	No	9	25.7%	8	7.0%	17	11.3%	*p* = 0.005 **	
TMJ disorders	Yes	20	57.1%	80	69.6%	100	66.7%	Chi^2^ = 1.863	0.111
	No	15	42.9%	35	30.4%	50	33.3%	*p* = 0.172	
Muscular disorders	Yes	18	51.4%	55	47.8%	73	48.7%	Chi^2^ = 0.139	0.030
	No	17	48.6%	60	52.2%	77	51.3%	*p* = 0.709	
MC misalignment	Yes	26	74.3%	72	62.6%	98	65.3%	Chi^2^ = 1.615	0.104
	No	9	25.7%	43	37.4%	52	34.7%	*p* = 0.204	
TMJ pain	Yes	3	8.6%	27	23.5%	30	20.0%	Chi^2^ = 3.727	0.158
	No	32	91.4%	88	76.5%	120	80.0%	*p* = 0.054	
Dyshomeostasis	Yes	14	40.0%	56	48.7%	70	46.7%	Chi^2^ = 0.815	0.074
	No	21	60.0%	59	51.3%	80	53.3%	*p* = 0.367	
SSDS	Yes	23	65.7%	80	69.6%	103	68.7%	Chi^2^ = 0.185	0.035
	No	12	34.3%	35	30.4%	47	31.3%	*p* = 0.667	
Total		35	100.0%	115	100.0%	150	100.0%		

** highly statistically significant.

**Table 5:** Distribution of edentulism complications by edentulism extension in patients eligible for hybrid prosthetic therapy.

		Edentulism Category								Total		Pearson’s Chi-Squared Test (df = 3)	Cramer’s V
		EPE		RPE		CE		SE					
		N	%	N	%	N	%	N	%	N	%		
Bone resorption	Yes	45	52.9%	-	-	41	87.2%	9	100.0%	95	63.3%	Chi^2^ = 36.271	0.492 **
	No	40	47.1%	9	100.0%	6	12.8%	-	-	55	36.7%	*p* < 0.001 **	
Irregular ridge	Yes	20	23.5%	-	-	39	83.0%	3	33.3%	62	41.3%	Chi^2^ = 51.305	0.585 **
	No	65	76.5%	9	100.0%	8	17.0%	6	66.7%	88	58.7%	*p* < 0.001 **	
Long/thick frenum	Yes	-	-	-	-	12	25.5%	3	33.3%	15	10.0%	Chi^2^ = 28.487	0.436 **
	No	85	100.0%	9	100.0%	35	74.5%	6	66.7%	135	90.0%	*p* < 0.001 **	
Malocclusion	Yes	74	87.1%	3	33.3%	47	100.0%	9	100.0%	133	88.7%	Chi^2^ = 34.799	0.482 **
	No	11	12.9%	6	66.7%	-	-	-	-	17	11.3%	*p* < 0.001 **	
TMJ disorders	Yes	49	57.6%	-	-	42	89.4%	9	100.0%	100	66.7%	Chi^2^ = 36.505	0.493 **
	No	36	42.4%	9	100.0%	5	10.6%	-	-	50	33.3%	*p* < 0.001 **	
Muscular disorders	Yes	29	34.1%	-	-	38	80.9%	6	66.7%	73	48.7%	Chi^2^ = 36.389	0.493 **
	No	56	65.9%	9	100.0%	9	19.1%	3	33.3%	77	51.3%	*p* < 0.001 **	
MC misalignment	Yes	48	56.5%	-	-	41	87.2%	9	100.0%	98	65.3%	Chi^2^ = 34.638	0.481 **
	No	37	43.5%	9	100.0%	6	12.8%	-	-	52	34.7%	*p* < 0.001 **	
TMJ pain	Yes	27	31.8%	-	-	-	-	3	33.3%	30	20.0%	Chi^2^ = 22.353	0.386 **
	No	58	68.2%	9	100.0%	47	100.0%	6	66.7%	120	80.0%	*p* < 0.001 **	
Dyshomeostasis	Yes	40	47.1%	-	-	21	44.7%	9	100.0%	70	46.7%	Chi^2^ = 18.240	0.349 **
	No	45	52.9%	9	100.0%	26	55.3%	-	-	80	53.3%	*p* < 0.001 **	
SSDS	Yes	52	61.2%	-	-	42	89.4%	9	100.0%	103	68.7%	Chi^2^ = 35.402	0.486 **
	No	33	38.8%	9	100.0%	5	10.6%	-	-	47	31.3%	*p* < 0.001 **	
Total		85	100.0%	9	100.0%	47	100.0%	9	100.0%	150	100.0%		

** highly statistically significant.

**Table 6:** Distribution of edentulism complications by Kennedy classification in patients eligible for hybrid prosthetic therapy.

		Kennedy Class						Total		Pearson Chi-Squared Test (df = 2)	Cramer’s V
		I		II		IV					
		N	%	N	%	N	%	N	%		
Bone resorption	Yes	38	52.8%	9	42.9%	-	-	47	49.0%	Chi^2^ = 3.611	0.194
	No	54	47.2%	12	57.1%	3	100.0%	49	51.0%	*p* = 0.164	
Irregular alveolar ridge	Yes	17	23.6%	3	14.3%	-	-	20	20.8%	Chi^2^ = 1.672	0.132
	No	55	76.4%	18	85.7%	3	100.0%	76	79.2%	*p* = 0.433	
Abnormal frenulum	Yes									-	-
	No	72	100.0%	21	100.0%	3	100.0%	96	100.0%	-	
Malocclusion	Yes	61	84.7%	15	71.4%	3	100.0%	79	82.3%	Chi^2^ = 2.638	0.166
	No	11	15.3%	6	28.6%	-	-	17	17.7%	*p* = 0.267	
TMJ disorders	Yes	40	55.6%	6	28.6%	3	100.0%	49	51.0%	Chi^2^ = 7.708	0.283 *
	No	32	44.4%	15	71.4%	-	-	47	49.0%	*p* = 0.021 *	
Muscular disorders	Yes	28	38.9%	3	14.3%	-	-	31	32.3%	Chi^2^ = 5.978	0.250 *
	No	44	61.1%	18	85.7%	3	100.0%	65	67.7%	*p* = 0.050 *	
MC misalignment	Yes	41	56.9%	9	42.9%	-	-	50	52.1%	Chi^2^ = 4.659	0.220
	No	31	43.1%	12	57.1%	3	100.0%	46	47.9%	*p* = 0.097	
ATM pain	Yes	15	20.8%	9	42.9%	3	100.0%	27	28.1%	Chi^2^ = 11.815	0.351 **
	No	57	79.2%	12	57.1%	-	-	69	71.9%	*p* = 0.003 **	
Dishomeostasis	Yes	34	47.2%	6	28.6%	-	-	40	41.7%	Chi^2^ = 4.539	0.217
	No	38	52.8%	15	71.4%	3	100.0%	56	58.3%	*p* = 0.103	
SSDS	Yes	37	51.4%	12	57.1%	3	100.0%	52	54.2%	Chi^2^ = 2.837	0.172
	No	35	48.6%	9	42.9%	-	-	44	45.8%	*p* = 0.242	
Total		72	100.0%	21	100.0%	3	100.0%	96	100.0%		

* statistically significant; ** highly statistically significant.

## Data Availability

Data is contained within the article.

## Abbreviations

The following abbreviations are used in this manuscript:

CE	complete edentulism
EPE	extended partial dentulism
MC	maxillo-cranial
Md	mandibular
Mx	maxillary
RPE	reduced partial edentulism
SE	subtotal edentulism
SSDS	stomatognathic system dysfunctional syndrome
TMJ	temporo-mandibular joint
